# Efficacy of drug regimens containing omadacycline in a murine model of *Mycobacterium avium* chronic lung infection

**DOI:** 10.3389/fmicb.2026.1821617

**Published:** 2026-05-29

**Authors:** Jean-Philippe Lanoix, Antoine Froment, Julia Delomez, Jessica V. Pierce, Alisa W. Serio, Morgane Choquet, Marie Perret, François Peltier, Cédric Joseph, Denis Chatelain, Claire Andréjak

**Affiliations:** 1 AGIR EA4294, Université de Picardie Jules Verne, Amiens, France; 2 Infectious Diseases Department, Amiens-Picardie University Hospital, Amiens, France; 3 Pneumology Department, Amiens-Picardie University Hospital, Amiens, France; 4 Paratek Pharmaceuticals, Inc., King of Prussia, PA, United States; 5 Pathology Department, Amiens-Picardie University Hospital, Amiens, France

**Keywords:** aerosol infection, animal model, antibiotic, *Mycobacterium avium*, non-tuberculous mycobacteria, omadacycline, tetracycline

## Abstract

*Mycobacterium avium* complex (MAC) is the most common non-tuberculous mycobacteria (NTM) and most frequent causative pathogen of NTM pulmonary infections. MAC is often associated with comorbidities, chronic infections, and high mortality rates. New treatment options are needed to increase treatment efficacy and tolerance. Omadacycline is a semisynthetic tetracycline-class drug with broad-spectrum activity, including against MAC *in vitro*. In an aerosol infection model of MAC pulmonary disease, we evaluated 9 treatment regimens that included omadacycline (OMC) alone or in dual, triple, or quadruple combinations with existing antibiotics: rifampin (RIF), clofazimine (CFZ), ethambutol (EMB), and clarithromycin (CLR). Treatment efficacy was assessed based on reduction of bacterial load in the lung and spleen over 4 months. OMC alone and OMC-RIF-EMB groups demonstrated efficacy starting at 2 months of treatment with a final reduction in bacterial burden in the lung of ~1 log_10_ and >4 log_10_, respectively, compared with untreated mice. Omadacycline was as efficacious as rifampin in the standard-of-care regimen (CLR-RIF-EMB) over the first 3 months. The most potent combination tested in this study was OMC-CLR-CFZ, with a >5 log_10_ reduction of bacterial burden in the lung. This is the first study demonstrating omadacycline efficacy against *M. avium* in mice alone and as part of a combination regimen. These data suggest that further evaluation of omadacycline for the treatment of MAC-PD is warranted.

## 1 Introduction

*Mycobacterium avium* complex (MAC) is the most frequently observed causative pathogen of non-tuberculous mycobacteria (NTM) infections, 80% of which are pulmonary. Patients with underlying respiratory diseases have suboptimal treatment outcomes, with frequent drug-related side effects (Kwon et al., 2019; Daley and Winthrop, 2020). Guideline-recommended standard-of-care (SOC) treatment of MAC pulmonary disease (MAC-PD) is a combination regimen of a macrolide, rifampicin, and ethambutol three times per week for at least 12 months after culture conversion; depending on disease severity, it may include more frequent dosing or the addition of amikacin (Daley et al., 2020; Daley and Winthrop, 2020). Macrolide-resistant strains require daily treatment with a combination of rifampin, ethambutol, and a choice of clofazimine, moxifloxacin, bedaquiline, or amikacin (Kwon et al., 2019; Daley et al., 2020). Despite these guideline recommendations, nonclinical studies have shown that the addition of rifampin does not add to the antimycobacterial effects that are observed with a dual macrolide-ethambutol combination, nor does rifampin suppress macrolide resistance (Schildkraut et al., 2023). This has led some experts to call for the removal of rifamycins from MAC-PD therapies. A randomized, controlled trial evaluating the efficacy of ethambutol-azithromycin vs. rifampin-ethambutol-azithromycin in patients with MAC-PD is ongoing (NCT03672630) and should assist in clarifying the role of rifampin in the treatment of MAC-PD (van Ingen et al., 2024). Additionally, alternative treatment options are limited in the setting of macrolide-resistant or recurrent/refractory disease, or when patients experience drug intolerance to the SOC treatment regimen (Daley and Winthrop, 2020). An estimated 70% of patients report treatment-related side effects, which may include hepatoxicity, ocular toxicity, gastrointestinal effects, tinnitus, and ototoxicity (van Ingen et al., 2021). This often leads to specific drugs being discontinued, which may contribute to the development of drug resistance. Optimization of current therapies, and ultimately the development of a new SOC treatment regimen and suitable alternative agents for MAC-PD, is critical to improve patient outcomes and quality of life.

Omadacycline is a semisynthetic derivative of tetracycline that is available in both oral and intravenous formulations and is currently approved by the United States Food and Drug Administration for the treatment of adults with community-acquired bacterial pneumonia (CABP) and acute bacterial skin and skin structure infections (ABSSSI) caused by susceptible microorganisms (Paratek Pharmaceuticals; Macone et al., 2014). Omadacycline has broad spectrum *in vitro* activity, including activity against two of the most common NTM species, *M. avium* and *M. abscessus* (Brown-Elliott and Wallace, 2021; Chapagain et al., 2022). In a hollow-fiber model, omadacycline demonstrated dose-dependent efficacy against *M. avium*, and high-dose monotherapy was as effective as the SOC regimen of clarithromycin-rifampin-ethambutol (Chapagain et al., 2022). In time-kill experiments with *M. avium*, omadacycline demonstrated highly synergistic effects when combined with amikacin, as well as enhanced activity with clarithromycin, ethambutol, and clofazimine (Mudde et al., 2026). Additionally, omadacycline prevented the emergence of resistance to both amikacin and clarithromycin in this model. *In vivo* activity of omadacycline vs. NTM has been demonstrated against *M. abscessus* in a mouse model of pulmonary infection; however, such data are lacking for *M. avium* (Lanoix et al., 2020; Nicklas et al., 2022; Rimal et al., 2023).

The aim of this study was to determine the efficacy of omadacycline alone or in combination with current SOC drugs, including clarithromycin, rifampin, ethambutol, and clofazimine, in an aerosol murine model of MAC-PD.

## 2 Materials and methods

### 2.1 Mycobacterial strain

*M. avium* strain Chester (MAC 101; American Type Culture Collection [ATCC] 700898) was grown to log-phase in Middlebrook 7H9 broth (Difco, Detroit, MI) with 10% (vol/vol) oleic acid-albumin-dextrose (OADC; Becton Dickinson, Le Pont-de-Claix, France) and 0.05% (vol/vol) Tween 80 (Sigma, St. Louis, MO). *M. avium* cultures were incubated for 4 weeks at 37 °C prior to use. This strain was mouse-passaged (Dickinson et al., 1992) and maintained at −80 °C.

### 2.2 Antimicrobials

Rifampin (Sigma-Aldrich, Saint-Quentin-Fallavier, France) and ethambutol (Sigma-Aldritch) were prepared in distilled water. Clarithromycin (Arrow Génériques Lyon, France) was solubilized in 10% absolute ethanol and then in distilled water. Clofazimine (Sigma-Aldrich) was prepared in 0.05% agarose. Omadacycline (Paratek Pharmaceuticals, Inc., PA, USA) was prepared in sterile phosphate-buffered saline (PBS). All stock solutions were stored at 4 °C for up to 1 week.

### 2.3 Aerosol infection with *M. avium*

BALB/c female 6-week-old mice were purchased from Charles River, Saint-Germain-Nuelles, France. All animal procedures were approved by the Animal Care and Use Committee of Amiens Picardy Jules Verne University. Mice were infected via inhalation exposure (Glas-Col, Terre Haute, IN) with 10 mL pure culture of *M. avium* strain Chester.

### 2.4 Study design

Mice were aerosol-infected in two biological replicates with 9.09 log_10_ colony-forming units (CFU)/mL *M. avium* using the inhalation exposure system. After infection, mice were randomized into nine treatment groups and one untreated group. At 28 days post infection, treatment was initiated 5 days/week for a total of 4 months with one of the following drug combinations: omadacycline; clarithromycin; omadacycline-clofazimine; omadacycline-clarithromycin; clarithromycin-rifampin-ethambutol (SOC); omadacycline-clarithromycin-ethambutol; omadacycline-rifampin-ethambutol; omadacycline-clarithromycin-clofazimine; or omadacycline-clarithromycin-rifampin-ethambutol. Drug doses were selected based on human equivalent exposures (Supplementary Table S2). An omadacycline dose of 15 mg/kg was selected based on pharmacokinetic (PK) parameters previously reported in BALB/c mice that showed an area under the curve (AUC) h × μg/mL of 11.36 for this dose compared to that in humans of 11.16 at steady state after administration of the 300 mg oral omadacycline dose (Paratek Pharmaceuticals, 2021; Nicklas et al., 2022). The remaining drug regimens examined, specifically 10 mg/kg rifampin, 25 mg/kg clofazimine, 100 mg/kg clarithromycin and 100 mg/kg ethambutol, have been previously demonstrated to be efficacious in murine models of *M. avium* infection (Lanoix et al., 2020; Almeida et al., 2009; Andréjak et al., 2015). Clofazimine, clarithromycin and ethambutol were administered in a total volume of 0.1 mL by esophageal cannula. Rifampin 0.1 mL was administered by gavage 1 h before administration of the other drugs to avoid possible interactions (Dickinson et al., 1992; Grosset et al., 1992). Omadacycline was administered in a volume of 0.2 mL via intraperitoneal route, as omadacycline lacks oral bioavailability in rodents (Data on file, Paratek Pharmaceuticals, Inc.).

Five untreated animals were sacrificed on the day of treatment initiation (D0) to determine CFUs at 1 month post infection, and five animals from both treated and untreated groups were sacrificed monthly thereafter.

### 2.5 Histopathology

In each group, the left lungs of two mice were resected for pathological examination. The lung was fixed in 10% buffered formalin (Sigma-Aldrich). Lung specimens were embedded in paraffin, and 4-μm-thick paraffin-embedded tissues were cut and stained with hematoxylin–eosin (H&E)–saffron and Ziehl–Neelsen stains.

### 2.6 Assessment of treatment efficacy

Treatment efficacy was assessed on the basis of right lung and spleen CFUs. Lungs and spleens were manually homogenized in sterile PBS. Serial dilutions of lung or spleen homogenates were plated on selective Middlebrook 7H11 agar plates enriched with 10% OADC plus antibiotics: 5% cycloheximide, 5% carbenicillin, 2.5% polymyxin B, and 2% trimethoprim (Sigma-Aldrich). Plates were incubated for 8 weeks at 37 °C. Four grams of charcoal per liter (0.4%) were added into the agar plates of treatment groups containing clofazimine and clarithromycin at months 2, 3 and 4. Growth on charcoal-containing plates vs. non-charcoal-containing plates was only compared for undiluted spleen samples at month 2.

### 2.7 Minimum inhibitory concentration testing

Drug susceptibility testing by broth microdilution was performed according to CLSI guidelines (M24 and M62). Inocula were prepared in sterile water and standardized using a nephelometer (0.5 McFarland standard), then diluted in the appropriate media to obtain a final solution of 5 × 10^5^ CFU/mL. Susceptibility testing was performed in the following culture media: cation-adjusted Mueller–Hinton broth (CAMHB) or Middlebrook 7H9 (without Tween 80 or glycerol), both supplemented with 5% OADC. Ninety-six well (U bottom) transparent polystyrene microplates (Thermo-Scientific, Illkirch, France) were incubated at 37 °C for 14 days of incubation with readings at 7, 10, and 14 days post inoculation. Two-fold drug dilutions were performed with a final volume of 100 μL/well. Omadacycline concentrations ranges of 1–64 mg/L were tested. Minimal inhibitory concentration (MIC) was tested in duplicate and determined by two independent investigators, defined as the lowest concentration at which a well remained visually clear. Ciprofloxacin was used as a control (MIC, 1–2 mg/L; Supplementary Table S1).

### 2.8 Assessment of the potential for treatment resistance

MICs of *M. avium* isolates collected from mice throughout the course of treatment were determined. Isolates were archived from treatment groups and stored at −80 °C until evaluated. MIC values were compared with the parent reference strain *M. avium*. Two isolates from each group at each time point (months 1, 2, 3, and 4) were frozen, resulting in a total of 80 isolates that were tested in duplicate (160 samples). CAMHB +5% OADC with drug was prepared fresh weekly with *M. marinum* ATCC 927 as a control per CLSI guidelines, and plates were read at 10 days of incubation.

### 2.9 Statistical analysis

**Figure 1:**
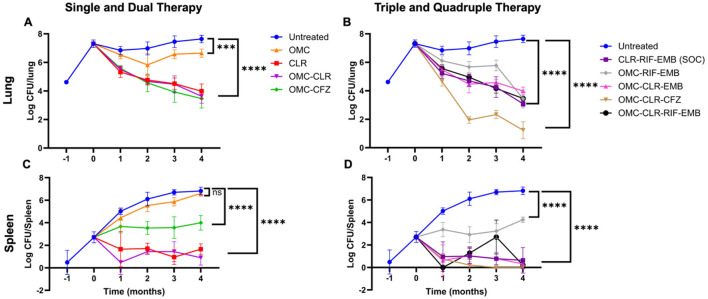
Total **(A, B)** lung and **(C, D)** spleen CFUs of 10 groups of *M. avium-*infected mice treated for 4 months. Data are presented as mean and standard deviation. Mixed effects analysis with Benjamini procedure: ***p < 0.001, ****p ≤ 0.0001. CFU, colony-forming unit; CFZ, clofazimine; CLR, clarithromycin; EMB, ethambutol; OMC, omadacycline; RIF, rifampin; SOC, standard of care.

**Figure 2:**
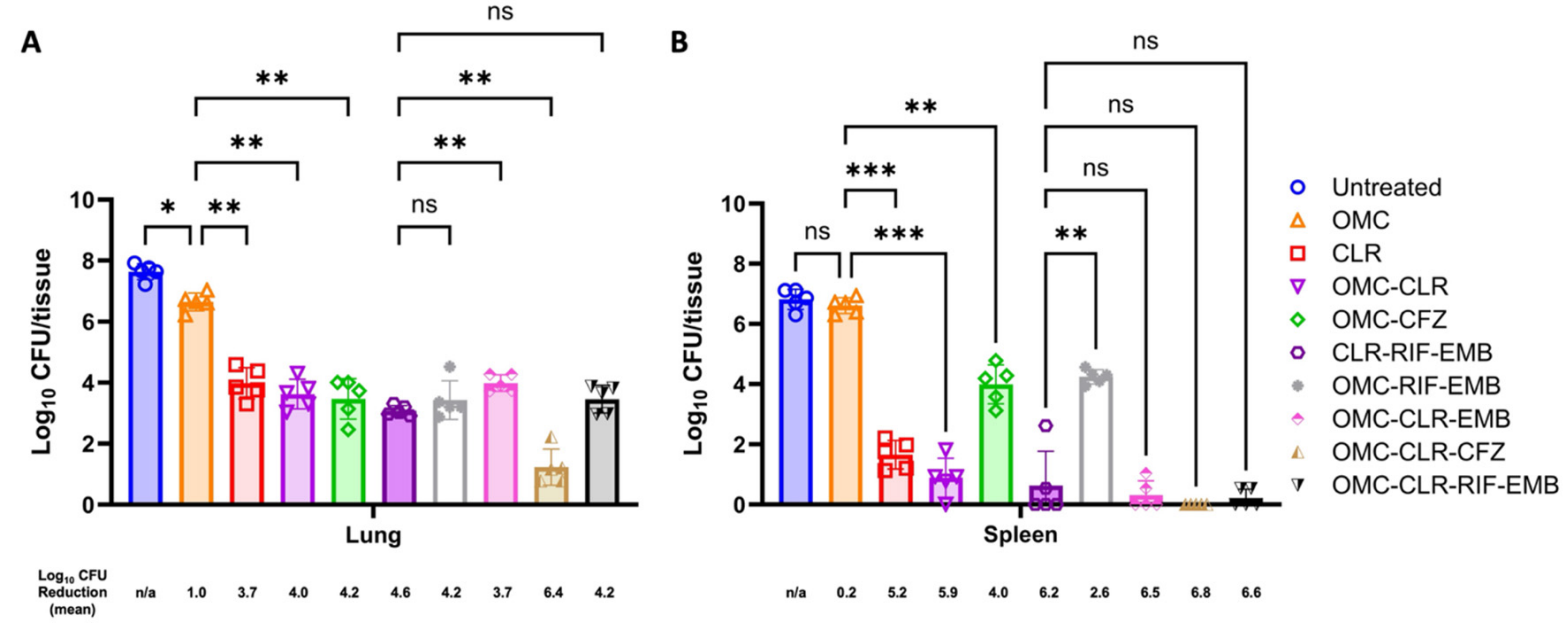
Mean reduction in bacterial tissue burden by treatment group after 4 months of treatment, compared with untreated mice, in the **(A)** lung and **(B)** spleen. Each symbol represents an individual animal; mean and standard deviation are shown. One-way ANOVA with Holm–Šidák multiple comparison test: *p < 0.05, **p < 0.01, ***p < 0.001. CFU, colony-forming unit; CFZ, clofazimine; CLR, clarithromycin; EMB, ethambutol; ns, not significant; OMC, omadacycline; RIF, rifampin.

CFU counts (*x*) were log transformed as log_10_(*x* + *1*) before analysis. To account for left lung loss, a 0.3 correction in CFU count was performed (Lanoix et al., 2015). The mean log_10_ CFU/lung ± the standard deviation (SD; *n* = 4–5 tissues per group per timepoint, independently plated, representing biological replicates) of *M. avium* was assessed throughout the study. Samples were plated as two technical replicates in a dilution series, and CFUs were averaged prior to calculating the mean log_10_ CFU per group. To compare CFU/organ between groups over time, a mixed-effects model with Benjamini procedure was used due to missing values (Figure 1) or repeated measure one-way analysis of variance (ANOVA) with Holm–Šidák multiple comparison test (Figure 2; *p* = 0.05 for significance) was performed (Prism GraphPad v6.07, La Jolla, CA).

## 3 Results

Mice infected with *M. avium* were treated with omadacycline alone or in combination with other SOC drugs for MAC-PD (Figure 1). One day post infection, the mean log_10_ ± SD CFU count of *M. avium* in the lungs was 4.6 ± 0.1 for the untreated group. After 1 month, the mean increased to 7.3 ± 0.3, demonstrating that *M. avium* had established infection. The bacterial load increased to a maximum of 7.6 ± 0.3 at month 4. In the spleen, prior to treatment, the mean CFU was 2.8 ± 0.5, which rose to 6.8 ± 0.3 at month 4 in untreated mice, demonstrating delayed dissemination of bacteria from the lung to the spleen. Individual animal CFU for both lung and spleen for all regimens at all timepoints are presented in Supplementary Tables S3 and S4, respectively.

### 3.1 Treatment efficacy

#### 3.1.1 One- and two-drug regimens

After 1 month, the omadacycline group had a log reduction (Δlog) of 0.3 compared with the untreated group (Figure 1A). All other groups demonstrated similar efficacy at this time point with a ~2-log reduction in bacterial burden. At 2 months, all treatment groups significantly reduced the bacterial burden compared with the untreated group, with a 1.2 Δlog for omadacycline alone. Omadacycline-clarithromycin and omadacycline-clofazimine performed similarly to the clarithromycin alone group (Δlog 1.3 and 1.2 vs. 1.5, respectively).

After 4 months, omadacycline treatment alone resulted in a significant decrease of 1 Δlog (*p* < 0.001) compared with untreated mice, whereas clarithromycin treatment alone resulted in a 3.7 Δlog (*p* < 0.0001; Figure 2A). There were no statistical differences between the effect of the clarithromycin and omadacycline-clarithromycin or omadacycline-clofazimine groups (Δlog of 3.7 vs. 3.6 and 4.2, respectively). The addition of either clarithromycin or clofazimine to omadacycline significantly increased efficacy in the lung.

Omadacycline alone was less efficacious in the spleen (Δlog 0.6) than in the lung (Figure 1C), whereas the addition of clofazimine to omadacycline effectively inhibited growth of *M. avium* after 1 month of treatment (Δlog 1.4). Clarithromycin treatment alone resulted in a Δlog of 3.4, which was improved with the addition of omadacycline to Δlog 4.5 after 1 month. Bacterial burden in the spleen was similar to the untreated group for the omadacycline group for months 2–4, and the other groups remained stable with a significant reduction in the omadacycline–clarithromycin and omadacycline–clofazimine groups compared with omadacycline alone (*p* < 0.05 and *p* < 0.001, respectively). The omadacycline-clarithromycin group had the most significant reduction in bacterial burden in the spleen, similar to clarithromycin alone, while the omadacycline-clofazimine combination was less efficacious. Charcoal was added to spleen samples at months 2, 3, and 4 to account for drug carryover: clofazimine- and clarithromycin-treated tissues had no or limited bacterial growth on plates without charcoal but had up to a two-log increase in bacterial CFUs in the presence of charcoal (Supplementary Table S5).

#### 3.1.2 Three- and four-drug combinations

The omadacycline–clarithromycin–clofazimine treatment group showed the largest reduction in bacterial burden compared with the untreated group, with a Δlog of 2.2 (*p* < 0.001) and a Δlog of 0.6 compared with the SOC at month 1 in the lung (Figure 1B). The omadacycline-rifampin-ethambutol treatment group had a Δlog of 0.7 compared with the untreated group at month 1, and was significantly less efficacious (*p* < 0.05) compared with the standard regimen. All other groups demonstrated a ~2-log reduction in bacterial burden at this time point.

After 2 months, all treatment groups significantly reduced the bacterial burden compared with the untreated group, with a Δlog of 5.0 for omadacycline–clarithromycin–clofazimine. Omadacycline–clarithromycin–clofazimine was the only treatment group significantly more efficacious than clarithromycin alone (Δlog 2.2). Omadacycline–clarithromycin–ethambutol and the SOC plus omadacycline performed similarly to the SOC, with a Δlog CFU of 0.2 and −0.3, respectively.

Omadacycline–rifampin–ethambutol treatment resulted in a reduction in bacterial load (Δlog CFU 4.2) comparable to clarithromycin–rifampin–ethambutol treatment (SOC, Δlog CFU 4.6) at the 4-month time point. Clarithromycin–rifampin–ethambutol was slightly more efficacious than omadacycline–clarithromycin–ethambutol (Δlog CFU 3.7; *p* < 0.05). The omadacycline–clarithromycin–clofazimine combination continued to demonstrate the highest efficacy out of all the combinations tested (Δlog 6.4 CFU; *p* < 0.001). Omadacycline plus the SOC was not significantly different than the SOC alone.

Similar to the lung, omadacycline–clarithromycin–clofazimine was the most potent combination tested in this study in reducing spleen mycobacterial burden, which decreased linearly from D0 to month 2 and then remained somewhat static through month 4 (Δlog CFU 6.2; *p* < 0.0001; Figures 1D, 2B). There were no significant differences between any of the other triple and quadruple combinations and the SOC at month 4, except for the omadacycline-rifampin-ethambutol treatment group (Δlog CFU 2.6 vs. untreated [*p* < 0.0001] and Δlog 3.6 vs. SOC [*p* < 0.01]; Figures 1D, 2B).

### 3.2 MIC values and assessment for resistance potential

The MIC values (mg/L) obtained for omadacycline against the parent strain *M. avium* ATCC 700898 varied depending on media type as well as length of incubation time (Supplementary Table 1). For example, in CAMHB + 5% OADC vs. 7H9 + 5% OADC, the omadacycline MIC values ranged from 2 to 4 mg/L vs. 16 to 32 mg/L, respectively, when incubated for 7 or for 10 days. After 14 days of incubation, omadacycline MIC values were ≥4-fold higher than those obtained at 7 or 10 days of incubation across both media types. In contrast, ciprofloxacin MIC values ranged from 1 to 2 mg/L, regardless of media type or length of incubation.

*M. avium* isolates from treatment groups passaged in mice underwent MIC testing to determine whether exposure to antibiotics induced drug resistance in this model. MICs of *in vivo* passaged isolates were compared with the parent strain (Supplementary Figure S1) in CAMHB +5% OADC and read after 10 days of incubation. The parent strain demonstrated MIC values of 4 to 64 mg/L for omadacycline, 0.25 to 1 mg/L for clarithromycin, 0.12 to 8 mg/L for rifampin, 4 to 16 mg/L for ethambutol, and 0.016 to 0.125 mg/L for clofazimine. The fold change in MICs for the passaged isolates compared with the parent strain were < 4-fold for clarithromycin, rifampin, and clofazimine. Omadacycline MICs increased 4- to 8-fold for the omadacycline-clarithromycin group at month 4, the omadacycline-clofazimine group at month 3, and the omadacycline–clarithromycin–ethambutol group at months 1, 2, and 3. However, the elevated fold changes in these groups were not consistent over time and did not persist through month 4, with the exception of the omadacycline–clarithromycin treatment combination, which showed increasing fold changes of MICs over time. However, no change in MIC was noted in the omadacycline alone treatment group.

### 3.3 Histopathology

**Figure 3:**
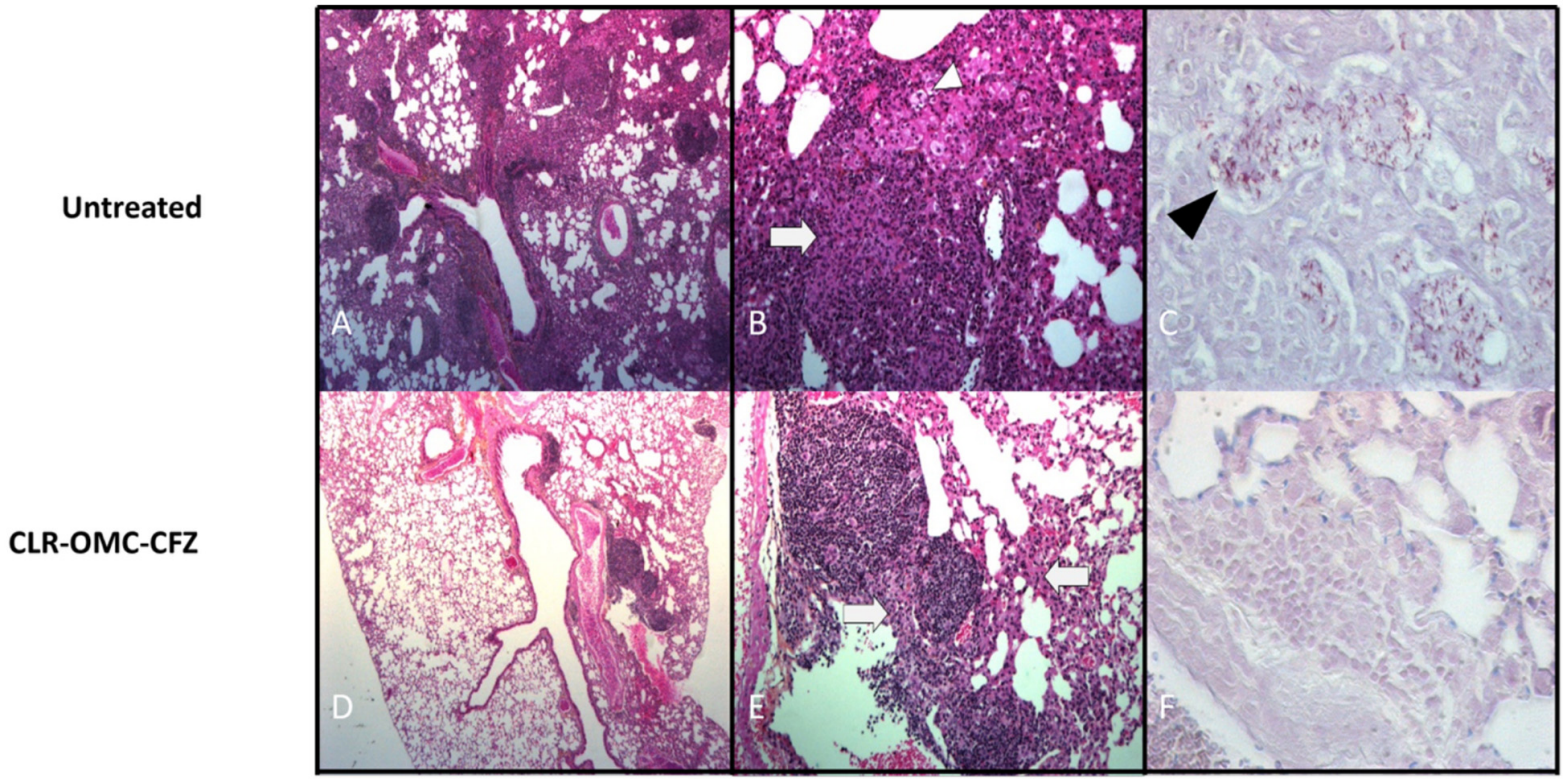
Inflammation in untreated and treated mice 4 months post infection with *M. avium*. Hematoxylin-eosin–saffron stain [2.5× **(A, D)**, 10× **(B, E)**] and Ziehl–Neelsen stain [**(C, F)** 40× ] left lung tissue after 4 months of infection with *M. avium:* Untreated, **(A–C)**; treated with OMC-CLR-CFZ, **(D–F)**. **(B)**: epithelioid granuloma (arrow), foamy macrophages (arrowhead). **(C)**: black arrow, intracellular acid-fast bacilli are shown in red. CFZ, clofazimine; CLR, clarithromycin; OMC, omadacycline.

Histopathology showed an increase in inflammation in the lungs over time in *M. avium*-infected mice. Untreated animals at 4 months post infection (Figure 3A) exhibited chronic inflammation concurrent with granuloma formation at 4 months post infection. Inflammatory infiltrates in pulmonary alveoli and the interstitium were observed, containing macrophages and lymphoid nodules (Figure 3A). Macrophages were observed in clusters in the pulmonary alveoli along with epithelioid granuloma, lymphocytes, plasma cells, and foamy macrophages (Figure 3B). Consistent with NTM infection, acid-fast bacilli were found in the presence of immune cells and intracellular bacteria were observed in intra-alveolar macrophages (Figure 3C).

In all treated mice, inflammation was reduced compared with the untreated group but was still present at low levels after 4 months of treatment. At month 4, inflammation and immune cells were still observed in the lung of the omadacycline–clarithromycin–clofazimine group, although with less alveolar filling (Figure 3D). This included focal clusters of lymphocytes and a few macrophages concurrent with very few to no visible acid-fast bacilli in alveolar macrophages (Figures 3E, F). No differences in the immune response between treatment groups were noted.

## 4 Discussion

Current treatment options for MAC-PD are poorly tolerated and have low efficacy rates; there is therefore an urgent need to investigate new drugs, especially oral treatments, for MAC. Identification of regimens with increased potency and fewer adverse effects compared with the current SOC is needed to improve clinical outcomes and shorten treatment duration in MAC-PD. Omadacycline is an oral and IV tetracycline-class antibiotic approved in the US for CABP and ABSSSI in adults, with no dose adjustments required for any patient group based on age, sex, race, weight, renal impairment, end-stage renal disease, or hepatic impairment (Leviton and Amodio-Groton, 2022). In addition, a recently completed Phase 2 randomized clinical trial against NTM-PD caused by *M. abscessus* demonstrated consistently favorable results when omadacycline was administered as a monotherapy for 3 months vs. placebo (Winthrop et al., 2026). Omadacycline was well tolerated; the most frequent treatment-emergent adverse event observed was gastrointestinal in nature, specifically nausea. While omadacycline has previously shown *in vitro* activity against *M. avium* and efficacy in a hollow-fiber model of MAC (Chapagain et al., 2022; Mudde et al., 2026), this is the first study to evaluate the *in vivo* efficacy of omadacycline against *M. avium* in mice at a human-equivalent dose (Nicklas et al., 2022). Omadacycline treatment alone demonstrated a significant reduction in lung bacterial burden (maximum Δlog 1.15) compared with the untreated control group. Clarithromycin monotherapy was also efficacious in this model, as expected, with a maximum Δlog 3.67 reduction in bacterial burden in the lung after 4 months of treatment. Although these results for omadacycline alone and clarithromycin alone should be interpreted with caution, given the potential selection of resistant mutants (Kwon et al., 2019), increased MIC values after monotherapy were not observed in this model but were observed for this combination at month 4. *In vitro* time-kill experiments did show that *M. avium* exhibited regrowth when clarithromycin and omadacycline were evaluated alone at 1 × MIC, with the MICs for clarithromycin increasing from 1 to ≥64 ug/mL (no data for omadacycline) (Mudde et al., 2026). Previous data demonstrated that the potential for the development of resistance to omadacycline was extremely rare for *M. abscessus*, but this has not been clearly demonstrated for *M. avium* (Rimal et al., 2023). Furthermore, no molecular mechanism for resistance to omadacycline in NTM has been identified. All omadacycline combination regimens demonstrated a significant reduction in lung and spleen bacterial burden after 4 months (Δlog 3.7 to 5.7 reduction compared with untreated control in the lung), which was similar to or exceeded the SOC regimen.

Importantly, none of the combination regimens that contained omadacycline demonstrated an obvious loss of efficacy relative to the comparators tested. The omadacycline–clarithromycin–clofazimine regimen was more efficacious than the current SOC and demonstrated the greatest reduction in MAC lung burden of all the combinations tested (Δlog 5.8 at month 4). The activity of omadacycline–clarithromycin–clofazimine was comparable to the previously reported activity of a clarithromycin–clofazimine–ethambutol triple regimen (Δlog 5.4 reduction at month 4) in the same mouse model (Lanoix et al., 2020). While ethambutol is an important component of the MAC-PD treatment regimen and contributes, along with clofazimine, to the suppression of macrolide resistance, ocular toxicity is a serious potential adverse effect (Griffith et al., 2005; Daley et al., 2020). These findings suggest that omadacycline–clarithromycin–clofazimine may be a promising alternative all-oral combination for MAC-PD. It is also important to highlight that clofazimine is not considered a first-line agent and is typically reserved for patients who have an intolerance to or whose causative pathogen is resistant to SOC agents (Daley et al., 2020). Clofazimine also may result in adverse effects such as skin discoloration or those that are gastrointestinal or neurological in nature, and close monitoring is imperative.

A major concern with any MAC regimen is protection against the development of macrolide resistance during treatment. While clofazimine may be an adequate substitute for rifampin in the SOC regimen, it has not been demonstrated to prevent macrolide resistance in the absence of ethambutol (Griffith and Aksamit, 2021). However, in a recently completed *in vitro* study, omadacycline alone prevented the development of clarithromycin resistance in *M. avium* (Mudde et al., 2026). Additional studies are needed to determine whether omadacycline can adequately prevent the development of macrolide resistance *in vivo* when combined with other SOC antibiotics or clofazimine.

Two combinations tested here that did not contain clarithromycin (omadacycline–rifampin–ethambutol and omadacycline–clofazimine) both exhibited similar reduction in bacterial burden to the current SOC regimen. The efficacy of omadacycline–rifampin–ethambutol combination would not be predicted by the activity of omadacycline alone and the limited additional killing of rifampin and ethambutol when combined with clarithromycin. Possible explanations for the activity of omadacycline–rifampin–ethambutol, which may warrant further study, include potential synergistic activity of omadacycline and rifampin and/or suppression of the development of resistance with combination therapy (Chapagain et al., 2022). Future studies could also explore whether the addition of a third non-macrolide agent could increase the activity observed with omadacycline–clofazimine, as there is a high need to identify effective regimens in the setting of macrolide resistance.

Strengths of this study include the use of a chronic pulmonary infection model in mice that most closely represents the pathology of clinical disease as shown by histopathological analysis, which confirmed the absence of extracellular acid-fast bacilli in lung tissue, as previously described (Andréjak et al., 2015), and high levels of lung inflammation in untreated mice with MAC-PD, although this did not lead to any outward clinical signs of disease. Granulomas were observed in the lungs of all groups and were sporadic in timing. Treatment led to a reduction of acid-fast bacilli observed in the lung that was consistent with the lung bacterial burden, and inflammation was persistent in treated mice despite the reduction in acid-fast bacilli. Tetracycline-class drugs, including omadacycline, have demonstrated anti-inflammatory properties, which could lead to decreased chronic inflammation of the lung in patients with long-term MAC-PD (Bryant and Stevens, 2022; Sanders and Beringer, 2024; Gomi et al., 2025).

Limitations of this study include a practical limit to the number of drugs and drug combinations that could be evaluated due to the number of time points and group size. In some cases, the lack of existing data for individual drugs limits the ability to objectively assess their contribution to the activity of a combination regimen. It would also be of interest to understand the interactions between omadacycline and amikacin, as amikacin is commonly included in MAC treatment regimens (Daley and Winthrop, 2020). Amikacin was not a viable option for inclusion in this mouse model because of toxicity concerns with the 4-month treatment duration. However, a recently completed *in vitro* time-kill study found that omadacycline was synergistic with amikacin and prevented the emergence of amikacin resistance in *M. avium* (Mudde et al., 2026). Additionally, the development of phenotypic resistance to macrolide monotherapy was not observed in this model after 4 months of treatment, despite clinical evidence demonstrating that the use of clarithromycin for 4 months led to selection of clarithromycin-resistant MAC strains (Chaisson et al., 1994). Therefore, a second drug is required to prevent macrolide resistance. It is unclear whether the slightly elevated fold changes in MIC values for omadacycline in the omadacycline-clarithromycin group are representative of clinical resistance patterns, but this result was unexpectedly not observed for omadacycline alone. Molecular analysis was also not performed but could be utilized in future studies as a second methodology to confirm lack or presence of known mutations that contribute to macrolide resistance, such as in the *rrl* gene. Typical treatment duration for *M. avium* is 12 months after culture conversion, so a longer treatment duration may be needed to observe this *in vivo*.

In addition, MIC values for omadacycline were highly variable and were dependent on media type and incubation time, a phenomenon that may make it difficult to determine if there was any decrease in susceptibility to omadacycline. For example, it is likely that the increases in omadacycline MIC values that were observed at month 4 in the combination regimen (omadacycline-clarithromycin) are due to inherent variability in the assay, especially because this was not observed with omadacycline treatment alone. As has been previously reported, omadacycline has demonstrated instability during prolonged incubation time in media, particularly for susceptibility testing of slowly growing mycobacteria. This is likely due to omadacycline being oxygen labile, similar to other members of the tetracycline class, i.e., tigecycline. The result of this instability may lead to falsely high MIC values (Shankar et al., 2022; Mudde et al., 2026). In this study, omadacycline MICs were read at day 10 as growth was too weak at day 7, and trailing and/or regrowth was observed at day 14. Similar observations of instability were seen for rifampin under the same conditions tested in this study (personal communication J. Lanoix), therefore limiting the utility of MICs in these cases to inform clinical use of these drugs. The use of fresh media (< 24 h/+4 °C storage following autoclaving) may potentially limit the negative impact of this phenomenon. Daily supplementation of drug could also mitigate this effect; however, it is labor intensive and risks contamination.

The interpretation of these results should also account for drug carryover. Long-term administration of drugs in chronic infection models such as this can lead to accumulation of drug in tissues. Subsequently, any drug still present in the tissue may lead to an underrepresentation of the true bacterial burden on agar plates. The phenomenon of drug carryover has been well described for clofazimine, but has yet to be determined for clarithromycin (Grosset et al., 2013). The intravenous infection models in which clarithromycin activity has been evaluated have high bacterial burden such that the effect of drug carryover would likely be masked. A comparison of agar plates with and without charcoal was performed with spleen samples and no growth was observed on plates without charcoal, while plates with charcoal had growth of bacteria from the groups treated with clarithromycin and clofazimine (data not shown). In future studies, a more thorough comparison between plain 7H11 and charcoal-containing plates should be performed to quantify carryover (Grosset et al., 2013). The qualitative analysis of bacteria and inflammation present in tissues supported the data measured for bacterial growth detected on charcoal-containing plates; however, quantitative scoring of both bacteria and inflammation would help interpret these data in future studies.

In conclusion, this is the first study demonstrating the efficacy of omadacycline as part of a combination treatment regimen for *M. avium* in a chronic pulmonary infection model in mice that closely represents the clinical disease of MAC-PD. These data suggest that further investigation of omadacycline against MAC-PD is warranted.

## Data Availability

The raw data supporting the conclusions of this article will be made available by the authors, without undue reservation.
