# Supplementary material for: Efficacy of drug regimens containing omadacycline in a murine model of Mycobacterium avium chronic lung infection
Source: Front Microbiol. 2026 May 29;17:1821617. doi: 10.3389/fmicb.2026.1821617 (PMC13260134; doi:10.3389/fmicb.2026.1821617)
Supplement: Supplementary file 1 [file Data_sheet_1.docx]

**SUPPLEMENTARY MATERIAL**

**Supplementary Table S1.** MICs (mg/L) of *M. avium* after 7, 10, and 14 days of incubation at 37 °C with either omadacycline or ciprofloxacin.

|  | **Omadacycline** | | **Ciprofloxacin** | |
| --- | --- | --- | --- | --- |
|  | CAMHB + 5% OADC | 7H9 + 5% OADC | CAMHB + 5% OADC | 7H9 + 5% OADC |
| 7 days | 2–4 mg/L | 16 mg/L | 1 mg/L | 1 mg/L |
| 10 days | 2–4 mg/L | 32 mg/L | 2 mg/L | 2 mg/L |
| 14 days | 16 mg/L | ≥64 mg/L | 2 mg/L | 2 mg/L |

Ciprofloxacin was included as a control.

CAMHB, cation-adjusted Mueller–Hinton broth; MIC, minimum inhibitory concentration; OADC, oleic acid-albumin-dextrose-catalase.

**Supplementary Table S2**. PK parameters of the human equivalent dose of antibiotics evaluated in mouse serum

| **Drug** | **Dose Evaluated** | **Humanized dose in BALB/c mice** | **AUC_0-24_ (h*µg/mL)** | **C_max_ (µg/unit)** | **T_max_ (h)** | **T_1/2_ (h)** |
| --- | --- | --- | --- | --- | --- | --- |
| Omadacycline^1,2^ | 15 mg/kg | 15 mg/kg | 11.36 | 4.16 | ND | 4.21 |
| Clarithromycin^3^ | 100 mg/kg | 75 mg/kg^a^ | 30.3 | 8.9 | ND | ND |
| Rifampin^4,5^ | 10 mg/kg | 10 mg/kg | 122.38^b^ | 5.67 | 1.5 | 13.75 |
| Ethambutol^6^ | 100 mg/kg | 100 mg/kg | 365.3^b^ | ~5 | ND | ND |
| Clofazimine^7^ | 25 mg/kg | 25 mg/kg | 19.3 | 0.43 | 1-4 | 87.46 |

AUC, area under the curve; BID, twice daily; C_max_, maximum concentration; ND, no data; PK, pharmacokinetic; T_1/2_, half life; T_max_, time to C_max_

^a^75mg/kg was the highest dose evaluated and the AUC_0-24_ corresponds to a human dose between 250 and 500 mg BID in humans

^b^ Data is shown for AUC_0-inf_

**1.** NUZYRA [package insert]. King of Prussia, PA: Paratek Pharmaceuticals, Inc. **2.** Nicklas D, et al. *Antimicrob Agents Chemother*. 2022;66: e0170421. **3.** Tagliabue C, et al. *J Antimicrob Chemother*. 2011;66:2323-2329. **4.** Hosagrahara V, et al. *Eur J Pharm Sci*. 2013;49:33-38. **5.** Stott KE, et al. *J Antimicrob Chemother*. 2018;73:2305-2313. **6.** Muliaditan M, et al. *Eur J Pharm Sci*. 2022;173:106163. **7.** Swanson RV, et al. *Antimicrob Agents Chemother*. 2015;59:3042-3051.

**Table S3: Per-mouse CFU/mL counts, Lung.**

|  |  |  |  | Log CFU/mL corrected (33%) | | | |
| --- | --- | --- | --- | --- | --- | --- | --- |
| **Day 0, Mouse#** | **Log CFU/mL** |  | **Treatment Group, Mouse#** | **Month 1** | **Month 2** | **Month 3** | **Month 4** |
| Untreated M1 | 7.31 |  | Untreated M1 | 7.00 | 6.90 | 7.23 | 7.76 |
| Untreated M2 | 7.48 |  | Untreated M2 | 6.70 | 7.09 | 7.01 | 7.65 |
| Untreated M3 | 7.47 |  | Untreated M3 | 6.72 | 6.24 | 7.93 | 7.64 |
| Untreated M4 | 7.44 |  | Untreated M4 | 6.54 | 7.32 | 7.62 | 7.22 |
| Untreated M5 | 6.86 |  | Untreated M5 | 7.26 | 7.35 | ND | 7.92 |
|  |  |  | CLR M1 | 4.94 | 4.20 | 4.68 | 4.59 |
|  |  |  | CLR M2 | 5.20 | 4.64 | 4.49 | 3.76 |
|  |  |  | CLR M3 | 5.93 | 5.02 | 4.84 | 3.30 |
|  |  |  | CLR M4 | 5.41 | 5.03 | 3.83 | 4.38 |
|  |  |  | CLR M5 | 5.12 | 4.92 | 4.71 | 3.85 |
|  |  |  | OMC M1 | 6.59 | 5.94 | 6.61 | 7.03 |
|  |  |  | OMC M2 | 6.56 | 4.48 | 6.87 | 6.61 |
|  |  |  | OMC M3 | 6.81 | 6.11 | 6.14 | 6.73 |
|  |  |  | OMC M4 | 6.15 | 6.57 | 6.67 | 6.65 |
|  |  |  | OMC M5 | 6.82 | 6.03 | ND | 6.22 |
|  |  |  | CLR-OMC M1 | 5.75 | 4.76 | 5.29 | 3.04 |
|  |  |  | CLR-OMC M2 | 5.57 | 4.49 | 4.49 | 3.67 |
|  |  |  | CLR-OMC M3 | 5.53 | 4.44 | 4.69 | 3.81 |
|  |  |  | CLR-OMC M4 | 5.53 | 4.83 | 3.74 | 3.30 |
|  |  |  | CLR-OMC M5 | 5.13 | 4.81 | 4.14 | 4.30 |
|  |  |  | CLZ-OMC M1 | 5.62 | 4.32 | 4.27 | 3.14 |
|  |  |  | CLZ-OMC M2 | 5.76 | 3.80 | 4.40 | 3.73 |
|  |  |  | CLZ-OMC M3 | 5.38 | 5.04 | 2.65 | 4.00 |
|  |  |  | CLZ-OMC M4 | 5.39 | 4.34 | 4.29 | 2.46 |
|  |  |  | CLZ-OMC M5 | 5.76 | 5.33 | 4.02 | 4.00 |
|  |  |  | CLR-RIF-EMB M1 | 4.86 | 4.32 | 4.71 | 3.20 |
|  |  |  | CLR-RIF-EMB M2 | 5.20 | 4.33 | 4.75 | 3.31 |
|  |  |  | CLR-RIF-EMB M3 | 5.76 | 4.99 | 4.26 | 2.97 |
|  |  |  | CLR-RIF-EMB M4 | 4.94 | 5.22 | 3.00 | 2.91 |
|  |  |  | CLR-RIF-EMB M5 | 5.38 | 4.61 | 4.56 | 3.02 |
|  |  |  | OMC-CLR-EMB M1 | 5.59 | 5.05 | 4.69 | 3.73 |
|  |  |  | OMC-CLR-EMB M2 | 5.42 | 3.58 | 4.40 | 4.27 |
|  |  |  | OMC-CLR-EMB M3 | 5.73 | 4.90 | 4.55 | 3.74 |
|  |  |  | OMC-CLR-EMB M4 | 4.94 | 4.10 | 4.39 | 3.90 |
|  |  |  | OMC-CLR-EMB M5 | 5.72 | 4.68 | 4.85 | 4.27 |
|  |  |  | CLZ-CLR-OMC M1 | 4.99 | 2.08 | 2.22 | 1.16 |
|  |  |  | CLZ-CLR-OMC M2 | 4.37 | 2.04 | 2.52 | 2.22 |
|  |  |  | CLZ-CLR-OMC M3 | 5.00 | 2.11 | 2.44 | 1.20 |
|  |  |  | CLZ-CLR-OMC M4 | 4.60 | 1.59 | 1.88 | 0.78 |
|  |  |  | CLZ-CLR-OMC M5 | 4.33 | ND | 2.58 | 0.78 |
|  |  |  | OMC-RIF-EMB M1 | 5.52 | 5.98 | 5.31 | 4.52 |
|  |  |  | OMC-RIF-EMB M2 | 6.34 | 5.93 | 6.20 | 3.20 |
|  |  |  | OMC-RIF-EMB M3 | 6.15 | 5.18 | 5.92 | 3.36 |
|  |  |  | OMC-RIF-EMB M4 | 6.15 | 5.30 | 5.67 | 3.20 |
|  |  |  | OMC-RIF-EMB M5 | 6.40 | 5.95 | ND | 2.88 |
|  |  |  | OMC-RIF-EMB-CLR M1 | 5.30 | 4.69 | 4.41 | 2.96 |
|  |  |  | OMC-RIF-EMB-CLR M2 | 5.26 | 5.07 | 3.94 | 3.82 |
|  |  |  | OMC-RIF-EMB-CLR M3 | 5.83 | 4.95 | 3.74 | 3.65 |
|  |  |  | OMC-RIF-EMB-CLR M4 | 5.68 | 5.26 | 4.18 | 2.98 |
|  |  |  | OMC-RIF-EMB-CLR M5 | 5.76 | 4.81 | 4.54 | 3.88 |

CFU, colony forming unit; CLR, clarithromycin; CLZ, clofazimine; EMB, ethambutol; Mx, mouse x; ND, no data; OMC, omadacycline; RIF, rifampin

**Table S4. Per-mouse CFU/mL counts, Spleen.**

| **Day 0  Group, Mouse#** | **Log CFU/mL** |  | **Group, Mouse#** | **Month 1** | **Month 2** | **Month 3** | **Month 4** |
| --- | --- | --- | --- | --- | --- | --- | --- |
| Untreated M1 | 2.78 |  | Untreated M1 | 4.92 | 6.40 | 6.78 | 6.30 |
| Untreated M2 | 2.55 |  | Untreated M2 | 4.85 | 6.23 | 6.35 | 7.11 |
| Untreated M3 | 2.25 |  | Untreated M3 | 4.95 | 5.13 | 6.87 | 6.86 |
| Untreated M4 | 3.50 |  | Untreated M4 | 4.82 | 6.07 | 6.80 | 6.70 |
| Untreated M5 | 2.44 |  | Untreated M5 | 5.53 | 6.70 | ND | 7.12 |
|  |  |  | CLR M1 | 0.00 | 1.41 | 0.54 | 1.98 |
|  |  |  | CLR M2 | 0.00 | 1.41 | 1.45 | 1.75 |
|  |  |  | CLR M3 | 3.48 | 2.49 | 1.20 | 1.13 |
|  |  |  | CLR M4 | 2.40 | 1.82 | 0.00 | 2.21 |
|  |  |  | CLR M5 | 2.40 | 1.41 | 1.56 | 1.20 |
|  |  |  | OMC M1 | 4.48 | 4.81 | 6.29 | 6.39 |
|  |  |  | OMC M2 | 4.57 | 5.16 | 6.06 | 6.30 |
|  |  |  | OMC M3 | 5.00 | 5.63 | 5.47 | 6.72 |
|  |  |  | OMC M4 | 3.10 | 6.15 | 5.66 | 6.94 |
|  |  |  | OMC M5 | 5.03 | 5.93 | ND | 6.70 |
|  |  |  | CLR-OMC M1 | 2.40 | 1.85 | 2.79 | 0.78 |
|  |  |  | CLR-OMC M2 | 0.00 | 1.32 | 1.41 | 0.93 |
|  |  |  | CLR-OMC M3 | 0.00 | 1.41 | 0.54 | 0.93 |
|  |  |  | CLR-OMC M4 | 0.00 | 1.41 | 1.64 | 0.00 |
|  |  |  | CLR-OMC M5 | 0.00 | 1.20 | 0.78 | 1.82 |
|  |  |  | CLZ-OMC M1 | 3.80 | 2.94 | 3.05 | 3.58 |
|  |  |  | CLZ-OMC M2 | 3.12 | 3.39 | 4.51 | 4.19 |
|  |  |  | CLZ-OMC M3 | 4.50 | 3.57 | 2.25 | 4.28 |
|  |  |  | CLZ-OMC M4 | 3.14 | 3.31 | 3.54 | 3.12 |
|  |  |  | CLZ-OMC M5 | 3.76 | 4.45 | 4.47 | 4.78 |
|  |  |  | CLR-RIF-EMB M1 | 2.40 | 0.78 | 0.93 | 0.00 |
|  |  |  | CLR-RIF-EMB M2 | 0.00 | 0.00 | 1.37 | 0.00 |
|  |  |  | CLR-RIF-EMB M3 | 2.40 | 1.61 | 1.04 | 0.54 |
|  |  |  | CLR-RIF-EMB M4 | 0.00 | 1.66 | 0.54 | 0.00 |
|  |  |  | CLR-RIF-EMB M5 | 0.00 | 1.04 | 0.00 | 2.62 |
|  |  |  | OMC-CLR-EMB M1 | 0.00 | 1.41 | 1.04 | 0.00 |
|  |  |  | OMC-CLR-EMB M2 | 0.00 | 1.98 | 0.78 | 0.00 |
|  |  |  | OMC-CLR-EMB M3 | 3.10 | 1.04 | 0.78 | 1.04 |
|  |  |  | OMC-CLR-EMB M4 | 0.00 | 0.78 | 0.54 | 0.00 |
|  |  |  | OMC-CLR-EMB M5 | 0.00 | 0.00 | 0.78 | 0.54 |
|  |  |  | CLZ-CLR-OMC M1 | 0.00 | 0.00 | 0.00 | 0.00 |
|  |  |  | CLZ-CLR-OMC M2 | 0.78 | 0.54 | 0.00 | 0.00 |
|  |  |  | CLZ-CLR-OMC M3 | 1.04 | 0.00 | 0.00 | 0.00 |
|  |  |  | CLZ-CLR-OMC M4 | 1.32 | 0.54 | 0.00 | 0.00 |
|  |  |  | CLZ-CLR-OMC M5 | 0.54 | 0.00 | 0.00 | 0.00 |
|  |  |  | OMC-RIF-EMB M1 | 3.80 | 3.94 | 3.53 | 4.10 |
|  |  |  | OMC-RIF-EMB M2 | 3.10 | 2.00 | 3.77 | 4.30 |
|  |  |  | OMC-RIF-EMB M3 | 3.98 | 2.74 | 3.33 | 4.32 |
|  |  |  | OMC-RIF-EMB M4 | 3.54 | 3.10 | 2.30 | 3.93 |
|  |  |  | OMC-RIF-EMB M5 | 2.40 | 2.81 | ND | 4.56 |
|  |  |  | OMC-RIF-EMB-CLR M1 | 0.00 | 1.53 | 0.00 | 0.54 |
|  |  |  | OMC-RIF-EMB-CLR M2 | 0.00 | 2.11 | 3.30 | 0.54 |
|  |  |  | OMC-RIF-EMB-CLR M3 | 0.00 | 1.04 | 3.40 | 0.00 |
|  |  |  | OMC-RIF-EMB-CLR M4 | 0.00 | 0.78 | 3.40 | 0.00 |
|  |  |  | OMC-RIF-EMB-CLR M5 | 0.00 | 0.93 | 3.40 | 0.00 |

CFU, colony forming unit; CLR, clarithromycin; CLZ, clofazimine; EMB, ethambutol; Mx, mouse x; ND, no data; OMC, omadacycline; RIF, rifampin

**Table S5: Comparison of CFUs from undiluted spleen samples plated on agar plates with and without charcoal**

| **CFU/mL** | **Month 2** | | **Month 3** | | **Month 4** | |
| --- | --- | --- | --- | --- | --- | --- |
| **Treatment Group, Mouse#** | **Plain** | **Charcoal** | **Plain** | **Charcoal** | **Plain** | **Charcoal** |
| CLR M1 | 0 | 5 | ND | 1 | ND | 19 |
| CLR M2 | 0 | 5 | ND | 3 | ND | 11 |
| CLR M3 | 0 | 62 | ND | 3 | ND | 3 |
| CLR M4 | 0 | 13 | ND | 0 | ND | 41 |
| CLR M5 | 0 | 5 | ND | 7 | ND | 1 |
| CLR-OMC M1 | 1 | 14 | 2 | 133 | ND | 1 |
| CLR-OMC M2 | 0 | 4 | 0 | 3 | ND | 0 |
| CLR-OMC M3 | 0 | 5 | 0 | 0 | ND | 0 |
| CLR-OMC M4 | 0 | 5 | 0 | 11 | ND | 0 |
| CLR-OMC M5 | 0 | 3 | 0 | 0 | ND | 16 |
| CLZ-OMC M1 | 61 | 91 | ND | 208 | ND | ∞ |
| CLZ-OMC M2 | ∞ | ∞ | ND | ∞ | ND | ∞ |
| CLZ-OMC M3 | ∞ | ∞ | ND | 33 | ND | ∞ |
| CLZ-OMC M4 | ∞ | ∞ | ND | ∞ | ND | ∞ |
| CLZ-OMC M5 | ∞ | ∞ | ND | ∞ | ND | ∞ |
| CLR-RIF-EMB M1 | 1 | 1 | ND | 1 | ND | 0 |
| CLR-RIF-EMB M2 | 0 | 0 | ND | 2 | ND | 0 |
| CLR-RIF-EMB M3 | 0 | 8 | ND | 0 | ND | 1 |
| CLR-RIF-EMB M4 | 0 | 9 | ND | 1 | ND | 0 |
| CLR-RIF-EMB M5 | 0 | 2 | ND | 0 | ND | 118 |
| OMC-CLR-EMB M1 | 1 | 5 | ND | 1 | ND | 0 |
| OMC-CLR-EMB M2 | 0 | 19 | ND | 1 | ND | 0 |
| OMC-CLR-EMB M3 | 0 | 2 | ND | 2 | ND | 1 |
| OMC-CLR-EMB M4 | 0 | 1 | ND | 0 | ND | 0 |
| OMC-CLR-EMB M5 | 0 | 0 | ND | 2 | ND | 0 |
| CLZ-CLR-OMC M1 | 0 | 0 | 0 | 0 | ND | 0 |
| CLZ-CLR-OMC M2 | 1 | 0 | 0 | 0 | ND | 0 |
| CLZ-CLR-OMC M3 | 0 | 0 | 0 | 0 | ND | 0 |
| CLZ-CLR-OMC M4 | 1 | 0 | 0 | 0 | ND | 0 |
| CLZ-CLR-OMC M5 | 0 | 0 | 0 | 0 | ND | 0 |
| OMC-RIF-EMB-CLR M1 | 0 | 8 | ND | ND | ND | 1 |
| OMC-RIF-EMB-CLR M2 | 0 | 30 | ND | 400 | ND | 0 |
| OMC-RIF-EMB-CLR M3 | 0 | 2 | ND | 500 | ND | 0 |
| OMC-RIF-EMB-CLR M4 | 1 | 1 | ND | 500 | ND | 0 |
| OMC-RIF-EMB-CLR M5 | 0 | 2 | ND | 500 | ND | 0 |

∞, too many to count; CFU, colony forming unit; CLR, clarithromycin; CLZ, clofazimine; EMB, ethambutol; Mx, mouse x; ND, no data; OMC, omadacycline; RIF, rifampin


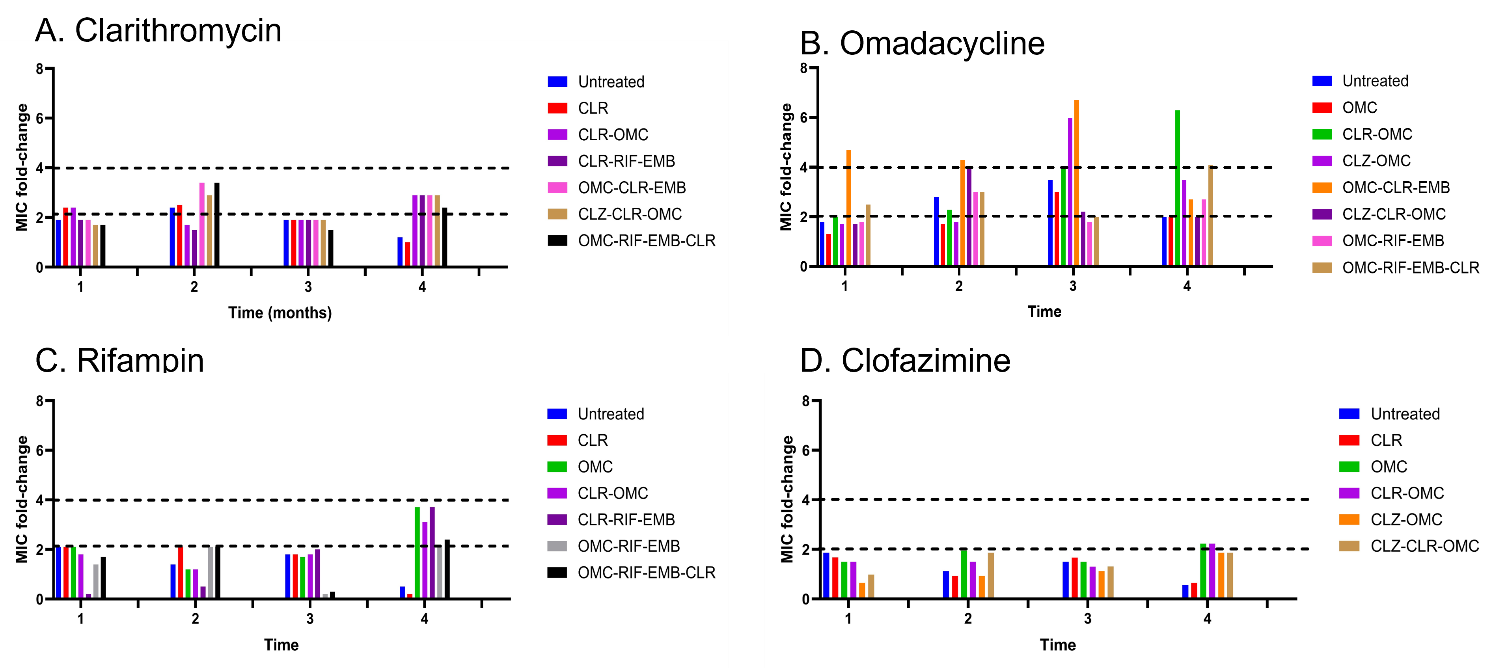


**FIGURE S1.** Fold change of MICs after passage *in vivo* compared with the *M. avium* parent strain. Time is shown in months. Fold change = passaged MIC/parent MIC. Isolates were grown in CAMHB+5% OADC and plates were read after 10 days of incubation. The parent strain was evaluated concurrently under identical conditions. CFZ, clofazimine; CLR, clarithromycin; EMB, ethambutol; MIC, minimum inhibitory concentration; OMC, omadacycline; RIF, rifampin. CAMHB, cation-adjusted Mueller–Hinton broth; MIC, minimum inhibitory concentration; OADC, oleic acid-albumin-dextrose-catalase.
